# A high-performance Cu–Al dual-ion battery realized by high-concentration Cl^−^ electrolyte and CuS cathode

**DOI:** 10.1038/s41598-022-23494-1

**Published:** 2022-11-04

**Authors:** Meina Tan, Yang Qin, Yiping Wang, Fazhi Zhang, Xiaodong Lei

**Affiliations:** 1 State Key Laboratory of Chemical Resource Engineering, Beijing University of Chemical Technology, Beijing, 100029 China; 2 Advanced Technology Department, RiseSun MGL, Inc., Beijing, 102299 China

**Keywords:** Electrochemistry, Green chemistry

## Abstract

We propose a new Cu–Al dual-ion battery that aqueous solution composed of LiCl, CuCl and AlCl_3_ (LiCuAl) is used as the electrolyte, CuS is used as the cathode of aqueous aluminum ion battery for the first time and copper foil is used as the anode. The assembled Cu–Al dual-ion battery yields a reversible capacity of 538 mA h/g at 200 mA/g, and exhibits longterm cycling stability of over 200 cycles with 88.6% capacity retention at 1000 mA/g. Above excellent performance is inseparable from the three components of LiCuAl electrolyte and electrode materials. The Al-storage mechanism of CuS is proposed that the S–S bond in CuS lattice interacts with aluminum ions during the aluminum storage process. In addition, the charging and discharging process does not cause irreversible damage to the S–S bond, thus Cu–Al dual-ion battery with CuS as cathode shows great cycle stability.

## Introduction

With the increasing demand for energy in human society, the energy storage and conversion has increasingly become a major issue that cannot be ignored. To meet these demands, current battery technologies, such as state-of-the-art lithium-ion batteries (LIBs) are the most widely used type of electrochemical battery for portable electronic devices^1,2^. In addition to technical problems such as potential safety hazards caused by lithium dendrites and volume expansion of electrode materials during charging and discharging, the scarcity of lithium-containing minerals and the high price of lithium compounds are also important factors that limit the application of lithium batteries^3,4^. Therefore, batteries using metal elements with high abundance in the earth (such as sodium, magnesium, zinc and aluminum) and non-flammable water-based electrolytes have received increasing attention^5,6^. Aluminum is the most abundant metal element in the earth and has active chemical properties. When used as battery electrode, it can undergo an electrochemical reaction of three-electron transfer. Among many metal anode electrodes, the theoretical mass specific capacity of aluminum is the second only to lithium (2980 mA h/g for Al, 3860 mA h/g for Li), and the volume specific capacity is nearly 4 times that of lithium (8040 mA h/cm^3^ for Al, 2062 mA h/cm^3^ for Li)^7,8^. Therefore, aluminum ion battery (AIB) is considered to be one of the most promising battery technologies.

The widely used electrolyte in AIB is the ionic liquid electrolyte prepared from AlCl_3_/1-ethyl-3-methylimidazolium chloride (EMIM^+^Cl^−^)^9–11^. Recently, an ionic liquid analog electrolyte, the mixture of AlCl_3_ and urea, has been reported for AIB^12–14^. However, the cost of ionic liquids is relatively high and urea-based cheap ionic liquid has a narrow stable electrochemical window. Moreover, these two electrolytes have high viscosity at room temperature that makes them difficult to achieve rapid ion migration and show low ionic conductivity^10^. In contrast, aqueous electrolytes tend to have high ion mobility and ionic conductivity, as well as the non-flammability and environmental friendliness, making aqueous AIBs become more and more concerned^15–21^. In recent years, aqueous batteries based on Water-in-Salt electrolytes have gradually emerged^22–24^. Many researchers have used high concentration of Al(OTF)_3_ electrolyte (Al-WISE) in aqueous AIB^18,21^ and have achieved certain results, but the high price of highly concentrated Al(OTF)_3_ electrolyte hinders its application.

In addition, the cathode material is also an important factor affecting the performance of AIB. Since Al^3+^ carries three positive charges, it has a strong electrostatic interaction with the surrounding atoms when they are embedded in the structure of the cathode material, resulting in the poor diffusion rate of Al^3+^ and even the structural destruction of the cathode material^17,25,26^. The use of carbon materials, such as graphite, or Prussian blue analogs (PBAs) with an open frame structure can solve this problem well^17,26–28^. However, the capacity of graphite and PBAs is relatively low and cannot meet the practical requirements. CuS exhibits a high aluminum storage capacity in EMIM^+^AlCl_4_^−^ based ionic liquid electrolyte, and it is converted to Cu_2_S, resulting in the formation of Al_2_S_3_ during the discharge process^29^. However, the aluminum storage characteristics of CuS in aqueous electrolyte have not been found.

Herein, a new type of aluminum ion aqueous electrolyte consisting of LiCl, CuCl and AlCl_3_ (LiCuAl) is designed. High concentration of Cl^−^ was introduced into the electrolyte to stabilize AlCl_x_(H_2_O)_y_^3−x^. Cu–Al dual-ion battery was assembled with copper foil as anode, CuS as cathode and LiCuAl as electrolyte. The results show that CuS nanosheets exhibit a high aluminum storage capacity, and the structure of CuS dose not undergo significant damage during the charge and discharge process, which means that the aluminum storage mechanism of CuS in the aqueous electrolyte in this study is different from that in the ionic liquid electrolyte. At the same time, the dual-ion battery has good rate performance and cycle stability.

## Results and discussion

### Structure and morphology of CuS

**Figure 1:**
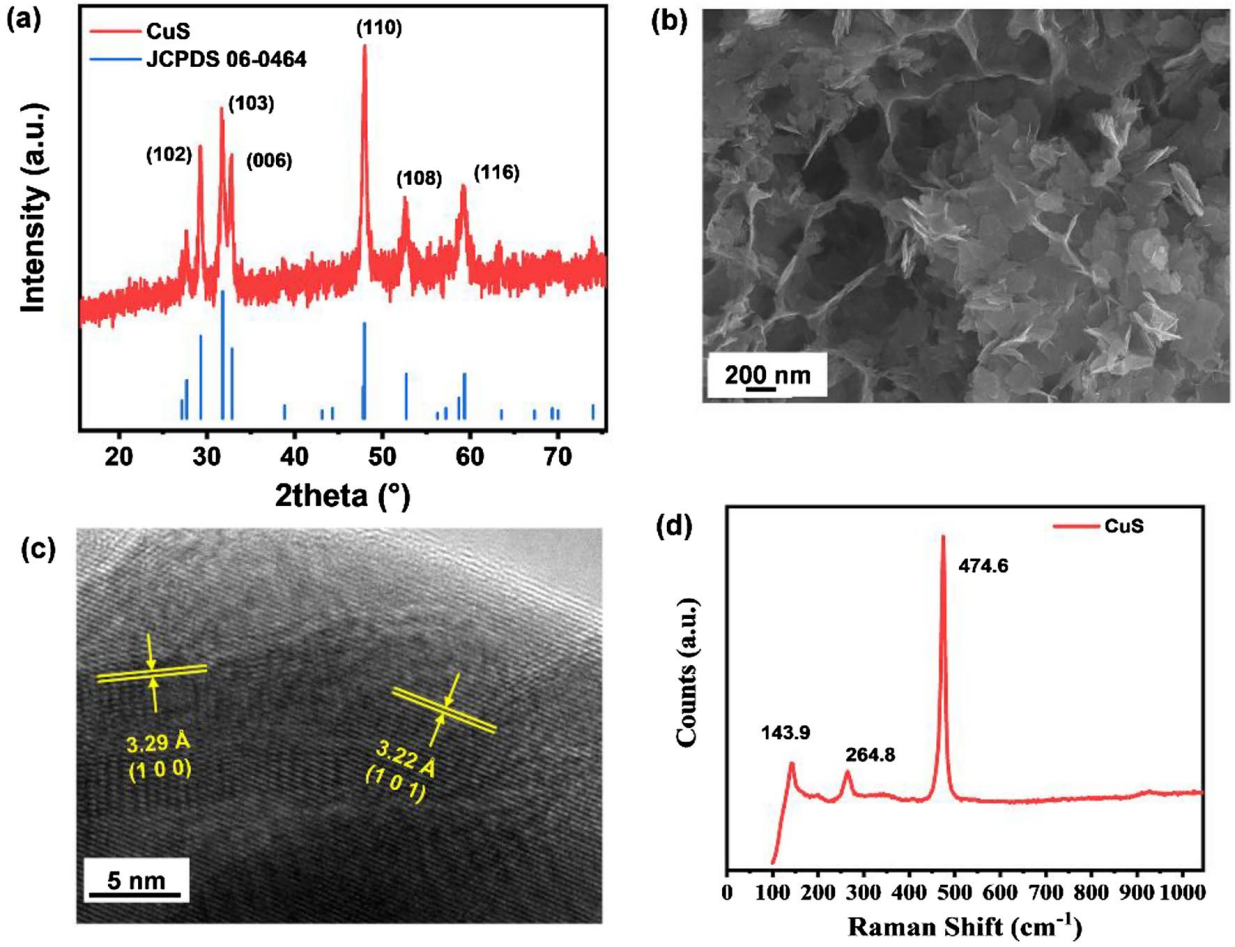
Structure and morphology of CuS: (**a**) XRD pattern, (**b**) SEM image, (**c**) TEM image and (**d**) Raman spectrum of CuS.

As shown in Fig. 1a, all X-ray diffraction (XRD) peaks of as-obtained sample are well indexed to hexagonal-phase covellite CuS (JCPDS No.06-0464, space group: P63/mmc, a = b = 3.792 Å, c = 16.344 Å)^29,30^, no impurity peaks were detected, indicating the successful preparation of CuS. As shown in Fig. 1b, the scanning electron microscope (SEM) image indicates that prepared CuS sample shows the morphology of the nanosheets. In the high-resolution transmission electron microscopy (HRTEM) image (Fig. 1c), two types of lattice fringes with distances of 3.29 and 3.22 Å are observed, corresponding to the (101)^31^ and (100)^32^ planes of covellite CuS, respectively. As illustrated by the Raman spectrum (Fig. 1d), three sharp peaks located at 143.9, 264.8 and 474.6 cm^−1^ match well with the reported CuS. The weak peaks at 143.9 and 264.8 cm^−1^ in the low frequency region are assigned to Cu–S vibrational stretching, and the strongest peak at 474.6 cm^−1^ is assigned to the stretching vibration mode of S–S^33,34^.

The existing forms of Cu and S on the surface of the CuS was analyzed by X-ray photoelectron spectroscopy (XPS). The Cu 2p spectrum (Fig. S1a) shows two pair peaks at 932.7/952.4 eV and 933.8/953.5 eV, which are attributed to 2p_3/2_/2p_1/2_ of Cu^+^ and Cu^2+^, respectively. Cu^+^ and Cu^2+^ are derived from the CuS unit cells of CuS_3_ and CuS_4_, respectively (Fig. S2)^29,34,35^. The existence form of reduced copper species was further demonstrated by Auger electron spectroscopy (AES, Fig. S1b), the peak at 570.2 eV in Cu LMM Auger spectrum indicates the existence of Cu^+^^36^. Figure S1c shows the XPS spectrum of S 2p, the peaks observed at 162.7 and 163.9 eV are attributed to the peaks of S 2p_3/2_ and S 2p_1/2_. The peak at 161.5 eV is attributed to the S_2_ unit in the unit cell of CuS^34,37,38^.

### Electrochemical Al-storage performance

**Figure 2:**
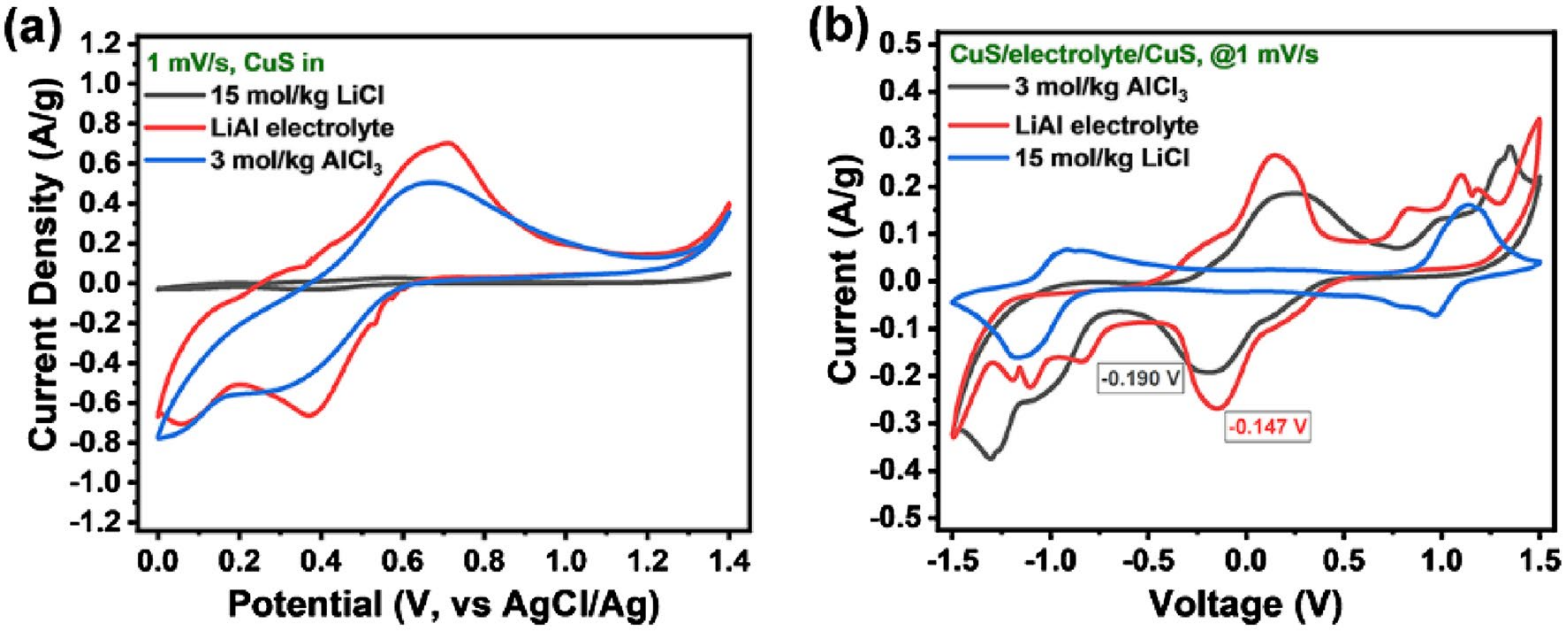
Electrochemical Al-storage performance of CuS electrodes: CV tests of (**a**) 3-electrode system and (**b**) symmetric battery in different electrolytes.

Figure 2A shows the cyclic voltammetry (CV) tests of CuS electrode in LiCl, LiAl (LiCl and AlCl_3_) and AlCl_3_ electrolytes, respectively. The CV curve of the CuS in the LiCl shows only a weak redox peak (Fig. 2a), indicating a slow electrochemical reaction. The appeared redox peak is related to the pseudo-capacitance behavior of CuS^39,40^. In the electrolytes containing AlCl_3_ (LiAl and AlCl_3_), both CV curves show strong oxidation peaks in the range of 0.5–0.8 V (vs AgCl/Ag) and reduction peaks at 0.4–0.05 V (vs AgCl/Ag), which is mainly related to the interaction between CuS and aluminum species. Similar results appear in the CV curves of symmetrical battery (Fig. 2b). When the voltage is low (− 0.5 to + 0.5 V), there is no redox peak appeared on the CV curve in the LiCl, while a pair of strong and symmetrical redox peaks appear in the electrolytes containing AlCl_3_. It is inferred that the CuS has a strong and reversible electrochemical reaction in the aluminum-containing electrolytes. When the voltage is higher than 1 V, the CV curves for all the three electrolytes showed redox peaks, which is related to the electrochemical oxidation/reduction of Cl^−^. The redox peak for the LiAl is the highest whether of three-electrode system or symmetric battery. The peak potential of symmetric battery in LiAl (− 0.147 V) is also lower than that in the AlCl_3_ (− 0.190 V), which means that the aluminum-related species in the LiAl are more likely to undergo electrochemical reactions on the surface of the CuS electrode.

It was reported that the main existing form of aluminum in AlCl_3_ aqueous solution is hydrated ion or hydroxylated ion^47,48^. In order to better understand the difference of the reaction mechanism and chemical kinetics of CuS in different electrolytes, and study the existence form of Al element in the electrolyte, the^27^Al nuclear magnetic resonance (NMR) spectra of electrolyte electrolytes containing Al species were obtained (Fig. S3). The^27^Al peak in the LiAl is at the higher field position (68 ppm) than that in AlCl_3_ (74 ppm)^41^. It shows that the addition of high concentration LiCl in LiAl promotes the coordination of Cl^−^ with Al^3+^. The electronegativity of chlorine is lower than that of oxygen, which makes the shared electrons between Al and Cl are more inclined to Al than those between Al and O, resulting in the more shielding of Al nucleus and the shift of ^27^Al peak to the higher field. Angell et al.^12^ reported that the existence of AlCl_4_^−^, Al_2_Cl_7_^−^ anions and [AlCl_2_·(urea)_n_]^+^ cations in the AlCl_3_/urea electrolyte when excess of AlCl_3_ was present_._ Coleman et al. also reported that the reaction of O-donor (dimethylacetamide, DMA) with AlCl_3_ produced a mobile liquid with high metal content, in which chlorides were replaced by neutral ligands to varying degrees, and neutral species coexisted with ionic species (DMA-AlCl_3_)^42^_._ Therefore, we believe that the ^27^Al peak in the electrolyte is attributed to the AlCl_x_(H_2_O)_y_^3−x^ complex ion. According to the NMR results, the x of the complex ion in LiAl is higher than that in AlCl_3_, and the y is lower than that in AlCl_3_. Therefore, AlCl_x_(H_2_O)_y_^3−x^ formed in LiAl is easier to remove the complex layer, resulting in the formation of aluminum ions that inserted into the crystal of the cathode electrode to participate in the electrode reaction. In addition, the broad peak shape in the ^27^Al NMR spectrum is due to the rapid chemical exchange in the solution^12,42^. Meanwhile, there is basically no difference in the NMR spectra of LiCuAl and LiAl electrolyte employed in the Cu–Al dual-ion battery.

In order to confirm the influence of the Li^+^ in the electrolyte on the electrochemical reaction of CuS in LiAl, LiCl is replaced by (CH_3_)_4_NCl. As shown in Fig. S4, the CV curves of the (CH_3_)_4_NCl-based electrolyte show oxidation peaks in the range of 0.5–0.8 V (vs AgCl/Ag) and reduction peaks at 0.4–0.05 V (vs AgCl/Ag) with the same peak positions as LiAl electrolyte. It means that the same electrochemical reactions of the CuS electrode occured in the two electrolytes. That is the electrochemical reaction of CuS in the LiAl is independent on Li^+^, but related to Cl^−^ based on the formation of AlCl_x_(H_2_O)_y_^3−x^. It is seen from Fig. S4 that although the electrolyte concentration is the same, the current density of the CV curve for the (CH_3_)_4_NCl-based electrolyte is lower than that for the LiAl at the same sweep rate. The reason is that the (CH_3_)_4_NCl-based electrolyte has the higher viscosity, which is not conducive to ion migration.

### Electrochemical performance of Cu–Al dual-ion battery

For battery, a piece of titanium foil coated with CuS (1 × 1 cm^2^) is used as cathode, and LiAl is used as electrolyte. Aluminum foil is used as anode firstly. However, the aluminum foil is corroded spontaneously in the LiAl, as shown in Fig. S5. It is inferred that the coordination between the high concentration of Cl^−^ and Al^3+^ promotes the reaction between metallic aluminum with water, leading to the dissolution of aluminum (Eqs. 1–3. The detailed analysis is shown in Note S1). In addition, hydrogen is generated in the spontaneous corrosion process (Eq. 2), which will cause safety hazards.

1 $${\text{Al}}^{{{3} + }} + {\text{3H}}_{{2}} {\text{O}} \rightleftharpoons {\text{Al}}\left( {{\text{OH}}} \right)_{{3}} + {\text{3H}}^{ + }$$

2 $${\text{2Al}} + {\text{6H}}^{ + } \rightleftharpoons {\text{2Al}}^{{{3} + }} + {\text{3H}}_{{2}}$$

3 $${\text{Al}}^{{{3} + }} + {\text{xCl}}^{ - } + {\text{yH}}_{{2}} {\text{O}} \rightleftharpoons {\text{AlCl}}_{{\text{x}}} \left( {{\text{H}}_{{2}} {\text{O}}} \right)_{{\text{y}}}^{{{3} - {\text{x}}}}$$

Metal Cu has the advantages of abundant natural content, low price, and the theoretical capacity of 844.3 mA h g^−1^^43^. Therefore, we use copper foil as the anode material to assemble batteries. In order to make the dissolution/deposition of the copper foil anode go smoothly, we added CuCl to the LiAl electrolyte to form LiCuAl. For comparison, the electrolyte containing 6.82 mol/kg (CH_3_)_4_NCl, 0.1 mol/kg CuCl and 1.64 mol/kg AlCl_3_ is also used as electrolyte, but a yellow precipitate is formed instead of a uniform and stable solution, while the LiCuAl electrolyte was a homogeneous and stable solution (Fig. S6).

**Figure 3:**
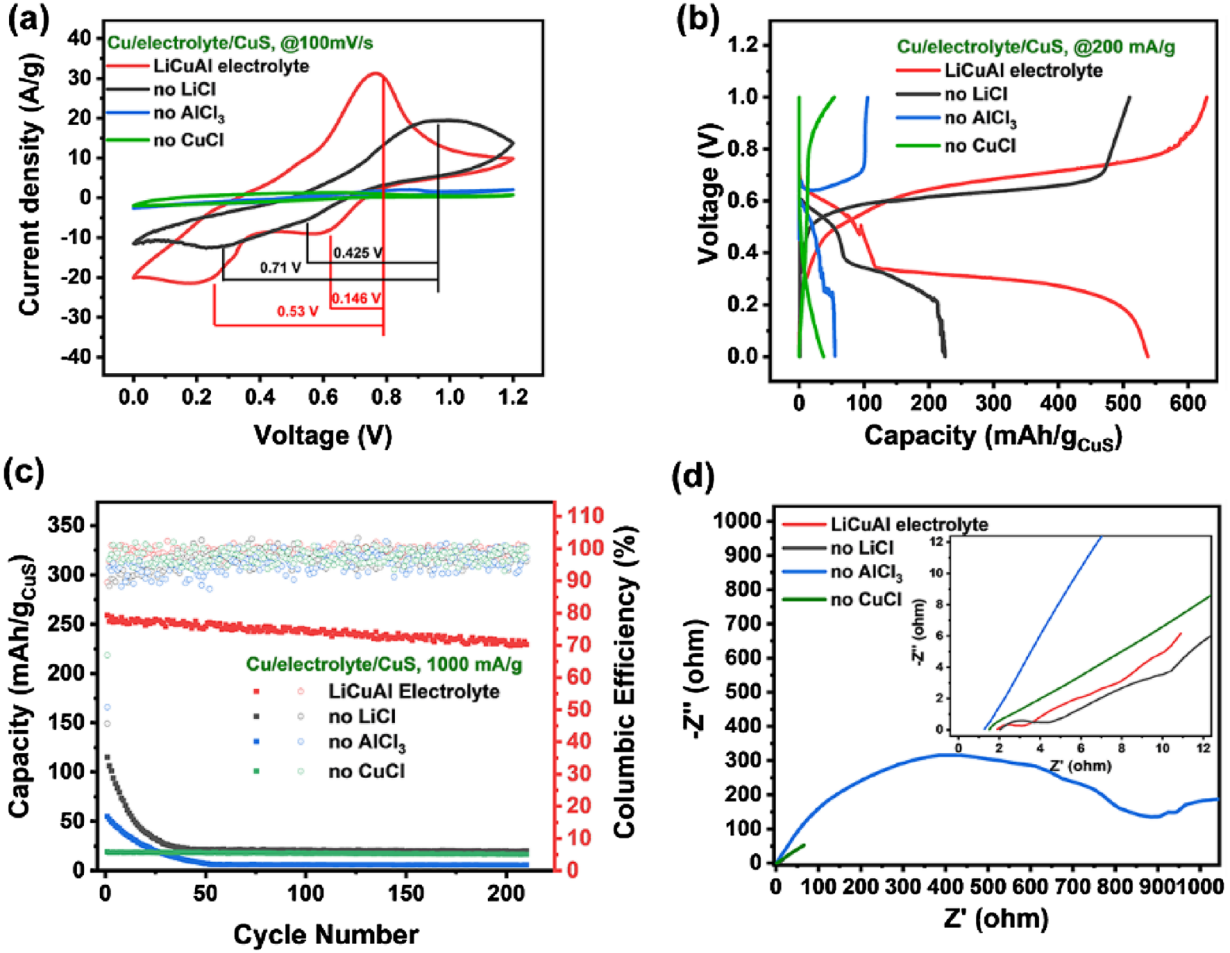
Electrochemical performance of Cu–Al dual-ion battery: (**a**) CV curves, (**b**) GCD curves, (**c**) cycling stability and (**d**) EIS of soft-packed dual-ion batteries.

The scheme of soft-packed Cu–Al dual-ion battery with copper foil as anode, CuS on titanium foil as cathode and LiCuAl as electrolyte is shown in Fig. S7. Figure 3a shows the CV curves of CuS in pouch cells with different electrolytes to study the electrochemical behavior. The Cu–Al dual-ion battery with LiCuAl as electrolyte shows the most significant redox peak and highest peak current. The oxidation peak at 0.77 V is related to the dealumination reaction of the cathode electrode, while the two reduction peaks at 0.62 and 0.24 V are related to the aluminum insertion process of CuS. The two reduction peaks coincide with the two discharge plateaus in the GCD curve, which may be caused by the reduction of Cu and S–S bond^32^. When LiCl is not in LiCuAl, the current density is significantly reduced, indicating that the high concentration of Cl^−^ in the LiCuAl effectively promotes the electrode reaction. In addition, the voltage gaps in LiCuAl electrolyte are 0.146 V and 0.53 V, respectively, which are smaller than those in LiCl-removed electrolytes (0.425 V and 0.71 V), indicating the higher electrochemical activity and better reaction kinetics of CuS in LiCuAl electrolyte^44,45^. There is no obvious redox peak on the CV curve when there is no addition of AlCl_3_ or CuCl, and the current density is extremely low, indicating that both Al^3+^ and Cu^+^ participate in the electrochemical reaction on the electrode.

The galvanostatic charge–discharge (GCD) test results of Cu–Al dual-ion battery with different electrolyte are shown in Fig. 3b. At current density of 200 mA/g, the battery with LiCuAl as electrolyte delivers a charging capacity of 629 mA h/g with corresponding discharging capacity of 538 mA h/g, giving a coulombic efficiency (CE) of 85.5%. When LiCl is not in the electrolyte, the charging capacity is 509 mA h/g, but only 224 mA h/g of discharging capacity and 44.0% of CE are obtained, indicating that CuS has high Al-storage performance. Meanwhile, we prepared electrolytes with different chloride ion concentrations and applied them to Cu–Al dual-ion batteries. The GCD curves are shown in Fig. S8. It can be seen that both the discharge capacity and the corresponding CE increase with the increase of LiCl concentration. As for the electrolyte without addition of AlCl_3_ or CuCl, the discharging capacities are only 55 and 37 mA h/g and the CEs are 52.4% and 68.5%, respectively, indicating that the aluminum and copper species have a positive effect on the energy storage process.

The high reversible capacity and excellent cycling behavior of the Cu–Al dual-ion battery are also exhibited in the rate capability. As shown in Fig. S9, the capacity gradually decreased with the increase of current density. After changing the current density from 1000 back to 200 mA g^−1^, the specific capacity reverts to 538 mA h g^−1^, indicating its high reversibility. The excellent rate capability is also demonstrated in Fig. 3c, soft-packed dual-ion battery still maintains 88.6% of the initial capacity after 200 cycles at current density of 1000 mA/g, which is the high-level value among reported for AIBs cathodes (shown in Tables S1 and S2). Meanwhile, CuS electrode still maintained nanosheet morphology after 200 cycles of testing, indicating its excellent structural stability for storage (Fig. S10). In the electrolyte without LiCl, the capacity quickly decays to 25% of the initial value within the initial 20 cycles. In the electrolyte without AlCl_3_, the capacity quickly decays to nearly zero, indicating that the reversible capacity of the dual-ion battery is mainly contributed by the electrochemical reaction between the aluminum species and CuS. In the electrolyte without CuCl, the capacity of the battery remains basically constant during 200 cycles, but its specific capacity is only about 10% of that in the LiCuAl.

To better understand the electrode reaction kinetics when using different electrolytes, the electrochemical impedance spectroscopy (EIS) of the battery was tested as shown in Fig. 3d^45^. The battery using LiCuAl electrolyte has the smallest impedance (0.8589 Ω), which means efficient charge transfer between the electrode and electrolyte. The impedance curve of the battery using the electrolyte without LiCl is similar to that of the battery using LiCuAl, but shows the larger charge transfer resistance (1.567 Ω). Furthermore, the steep slope of the cells using the LiCuAl electrolyte demonstrated lower diffusion resistance than the cells without using LiCl electrolyte. Therefore, the introduction of LiCl into the electrolyte can effectively reduce the charge transfer resistance, promote the charge transfer between the electrode and the electrolyte, and accelerate the diffusion kinetics of Al ions. At the same time, the battery using electrolyte without AlCl_3_ shows the great charge transfer resistance (390.1 Ω), indicating that Li^+^ and Cl^−^ are difficult to react with CuS. In other words, the energy storage performance of CuS in Cu–Al dual-ion batteries is mainly derived from the aluminum storage capacity of CuS. When the electrolyte without CuCl is used, the EIS is basically straight and its inclination angle is close to 45°, indicating that the reaction of battery at this time is mainly controlled by diffusion and basically no electrochemical reaction occurs.

**Figure 4:**
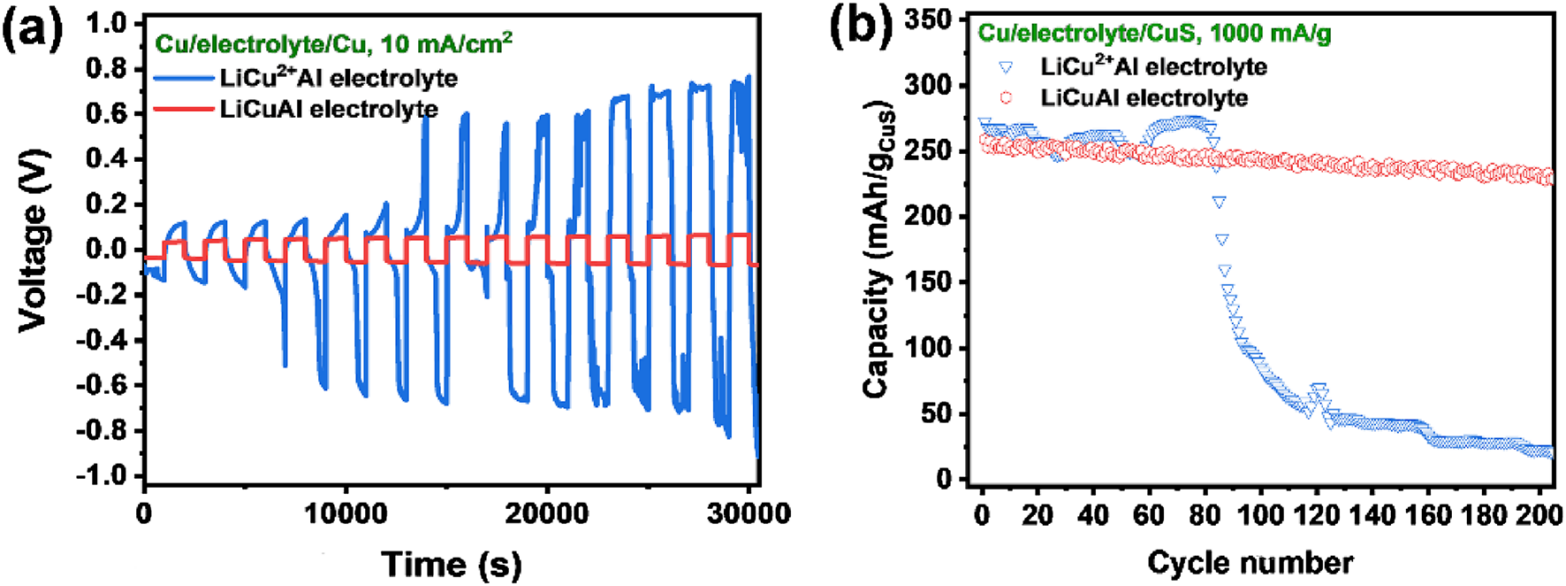
Electrochemical performance of copper anode and Cu–Al dual-ion battery in different valence copper salts: (**a**) cyclic charge–discharge tests of copper anodes. (**b**) Cycling stability of Cu–Al dual-ion battery.

The influence of electrolytes prepared with different valence copper salts on the electrochemical reaction of copper anode and the performance of the full battery was further explored. As shown in the cyclic charge–discharge curve of the symmetrical battery with copper foil as anode (Fig. 4a), the voltage difference of charge and discharge remains constant after the continuous test of 30,000 s in the LiCuAl prepared with CuCl, indicating that the relatively stable dissolution/deposition is achieved for the copper anode in LiCuAl. However, in the LiCu^2+^Al prepared with CuCl_2_·2H_2_O, the voltage difference of charge and discharge increases significantly after only three charge–discharge cycles (about 7000 s), indicating the reversibility of the dissolution/deposition reaction of Cu anode in LiCu^2+^Al is poor. Although the initial capacity is high (250–275 mA h/g) at 1000 mA/g, the capacity fluctuates greatly and there is a cliff-like attenuation after only 80 cycles (Fig. 4b), which is also due to the irreversible reaction in LiCu^2+^Al. In order to verify the influence of the valence state of copper in electrolyte on copper foil, two pieces of 1 × 1 cm^2^ copper foil is immersed in LiCuAl and LiCu^2+^Al overnight, respectively (Figure S11). It is found that the copper foil in the LiCuAl remains intact and bright, while the copper foil in the LiCu^2+^Al is obviously corroded. A large amount of Cl^−^ in the electrolyte complexes with Cu^2+^ to form CuCl_4_^2−^, and a neutralization reaction occurs between Cu and CuCl_4_^2−^ (Eq. (4)), which resulted in the corrosion of Cu foil. Therefore, the electrochemical dissolution/deposition of the copper foil in LiCu^2+^Al is irreversible. Based on above, the LiCuAl prepared with CuCl is more suitable for the battery.

4 $${\text{CuCl}}_{{4}}^{{{2} - }} + {\text{Cu}} \to {\text{2CuCl}}_{{2}}^{ - }$$

In addition, we investigated the stability of copper anode in LiCuAl electrolyte composed of CuCl. Because the copper foil was dissolved in the Cu^2+^ electrolyte (Fig. S11), we compared the change of the anode electrode copper foil before and after the battery performance test in the LiCuAl electrolyte formulated with CuCl, and found that it did not dissolve (Fig. S12). In order to observe the copper deposition morphology on the Cu foil, SEM was performed on the copper anode before and after the cycling performance reaction (Fig. S13a, b). Figure S13a shows that the surface of the copper foil is full of dense particles. After 200 cycles test, although the surface of the copper foil is no longer covered by the particles, a relatively compact and flat layer structure is still observed on the copper foil (Fig. S13b). The cross-section view image (Fig. S13d) shows that the copper foil after cycling shows a relatively uniform thickness with no visible dendrites. In addition, Fig. S14 shows the XRD patterns of the copper foil before and after the cycle test. The position of the diffraction peak remains unchanged, indicating that no new phase is generated. Therefore, the copper anode achieves stable dissolution/deposition in LiCuAl electrolyte in the presence of Cu^+^. The reactions taking place on the copper anode are as follows.

5 $${\text{Cu}}\left( {\text{s}} \right) + {\text{2Cl}}^{ - } \rightleftharpoons {\text{CuCl}}_{{2}}^{ - } + {\text{e}}^{ - }$$

The above results show that the three components (LiCl, AlCl_3_ and CuCl) of electrolyte, the copper anode, and the CuS cathode are all indispensable parts for Cu–Al dual-ion battery. Among them, AlCl_3_ provides aluminum ions for the cathode electrode reaction. The high concentration of LiCl provides a large amount of Cl^−^ for the coordination reaction with Al^3+^ to reduce the ion hydration layer and promote subsequent electrochemical reactions. The presence of CuCl enables the dissolution/deposition of the copper foil anode electrode to proceed smoothly. Therefore, the Cu–Al dual-ion battery exhibits high capacity and stability. Compared with commonly used AIB electrolytes, especially AlCl_3_/[EMIm]Cl, urea/AlCl_3_ and Al(OTF)_3_, the three components in LiCuAl are cheap, easy to obtain and safe.

### Mechanism investigation

**Figure 5:**
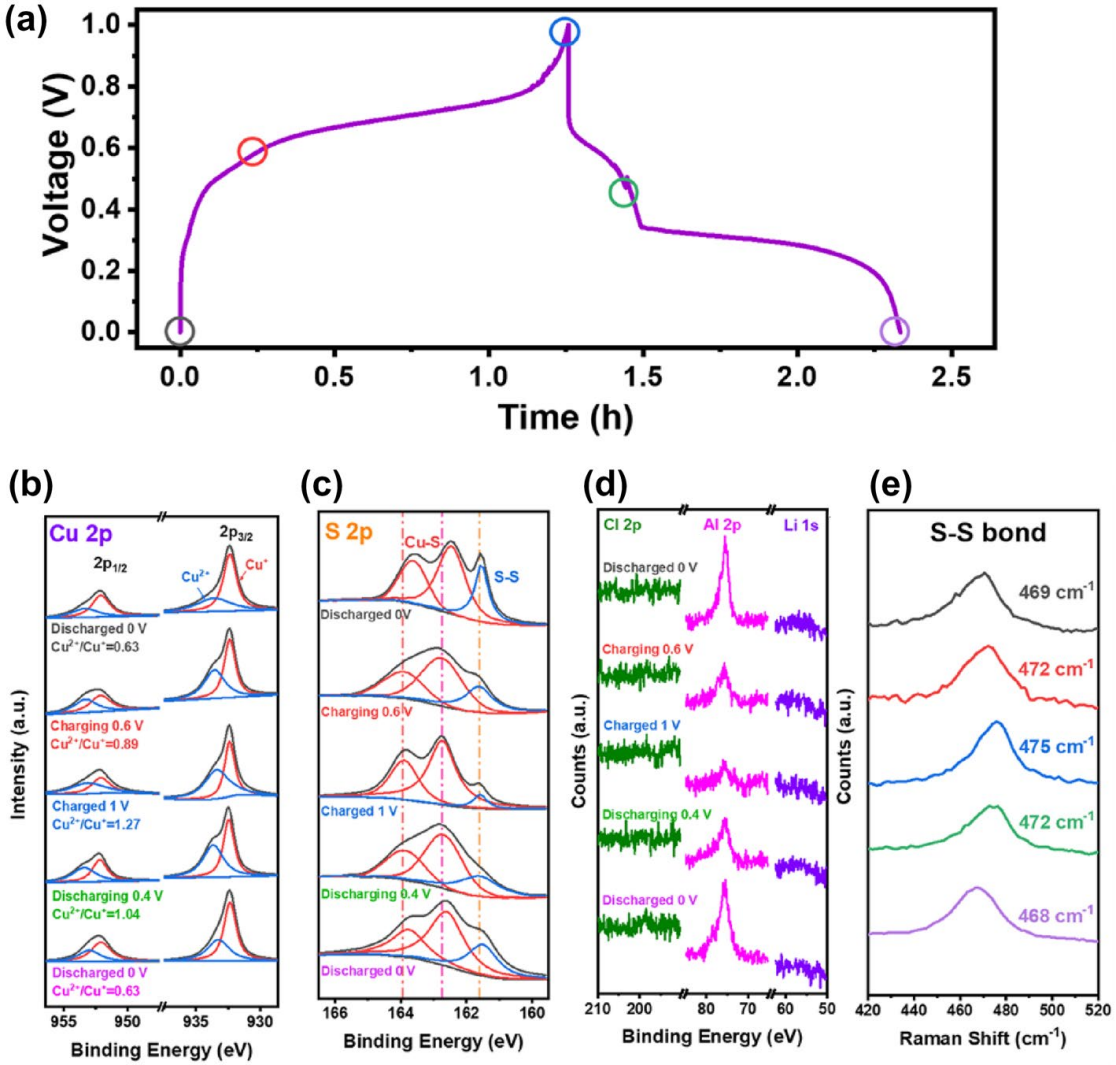
Ex-situ XPS and Raman of CuS under different charge/discharge states: (**a**) GCD profile, (**b**, **c**, **d**) ex-situ XPS spectra and (**e**) ex-situ Raman spectra.

CuS under different charge/discharge state (Fig. 5a) was characterized by the ex-situ XPS and Raman. As shown in Cu XPS spectra (Fig. 5b), the ratio of Cu^2+^/Cu^+^ increased from 0.63 to 1.27 during the charging process, indicating that a part of copper atoms is oxidized from CuS_4_ to CuS_3_ structure. The trend of the discharge process is the opposite that the ratio of Cu^2+^/Cu^+^ drops from 1.27 to 1.04 and finally returns to 0.63, indicating that the structural change of CuS is reversible. As shown in XPS spectra of S element (Fig. 5c), the valence state of S element in the Cu–S bond changes very little, and a relatively obvious difference is observed only between the fully discharged (0 V) and charged state (1 V). The peak intensity of the S–S bond weakens during the charging process and strengthens during the discharge process. Combined with the ex-situ XPS analysis of Cu (Fig. 5b), it is concluded that the S–S bond in the crystal lattice of CuS interacts with aluminum during Al-storage process. As shown in Fig. 5d, both the XPS peaks of Cl 2p and Li 1 s don’t appear in the entire test process, while the peak intensity of Al 2p shows a large decreasing with the charging process and increasing with the discharging process. It is indicated that under the test condition, neither Li^+^ nor Cl^−^ undergo insertion/detachment in the CuS lattice during electrochemical reaction. All the contribution of charge and discharge capacity comes from the interaction of aluminum-containing species in the electrolyte and CuS.

In the ex-situ Raman spectra (Fig. 5e), the characteristic peaks attributed to the S–S bond of CuS appears during the whole test, indicating that the CuS structure is maintained during the charge and discharge process without irreversible damage, which is contributed to the good cycle stability of CuS during the test. The characteristic peak position of the S–S bond has a slight blue shift during the charging process and returns to the initial value after the discharge process, and the wave number shift Δν is about 7 cm^−1^. In the charged state (1 V), the peak position of S–S bond is consistent with the CuS before reaction (Fig. 1d). During the discharge process, Al^3+^ enters into the CuS lattice, and the CuS_3_ structural unit is converted to CuS_4_, resulting the S–S bond obtains electrons, the electron cloud density increases, the bond level decreases and the reverse occurs during charging. As a result, the characteristic peak of the S–S bond in the Raman spectrum is blue-shifted during the discharge process, and red-shifted during the charging process. Therefore, the S–S bond in the CuS lattice interacts with Al^3+^ during the Al-storage process, which is independent of Li^+^ and Cl^−^ in the electrolyte. The charging/discharging process does not cause irreversible damage for the S–S bond. Therefore, the CuS nanosheets shows good cycle stability in the test.

## Conclusions

In this work, we propose a new type of Al^3+^ aqueous electrolyte for Cu–Al dual-ion battery with copper foil as anode and CuS on titanium foil as cathode. The three components of LiCuAl are all indispensable parts. When LiCuAl is used as electrolyte, as-assembled Cu–Al dual-ion battery exhibits the highest capacity of 538 mA h/g at 200 mA/g. The Al-storage mechanism is proposed that the Al-storage capacity of CuS is mainly contributed by the insertion/detachment reactions of Al^3+^ in the lattice of CuS, and the S–S bond is not irreversibly destroyed during the charge/discharge process. Therefore, the Cu–Al dual-ion battery exhibits excellent stability that retains initial capacity almost 88.6% after 200 cycles at 1000 mA/g.

## Methods

### Materials and reagents

CuCl_2_·2H_2_O (Sinopharm), Thiourea (Macklin), NaOH (Macklin), LiCl (Aladdin), (CH_3_)_4_NCl (Aladdin), CuCl (Sinopharm), anhydrous AlCl_3_ (Aladdin), and BaSO_4_ (Alfa) are analytical reagents and used without further purification. Titanium foil, Aluminum foil and Copper foil are purchased from Shengshida Metal Materials Industries.

### CuS preparation

CuS nanosheets were synthesized by grinding method according to our previous report^46^. 2 mmol CuCl_2_·2H_2_O and 4 mmol thiourea were mixed in an agate mortar and ground for 5 min until the color of mixture turned into light green. After that, 4 mmol NaOH was added and ground for 5 min until the mixture turn into a uniform black mash. The black mash was washed several times with deionized water and absolute ethanol, dried at 60 °C, then CuS was obtained.

### Electrolyte preparation

The electrolyte composition of single electrode test is 6.82 mol/kg LiCl and 1.64 mol/kg AlCl_3_ in deionized water, which named as LiAl electrolyte. For the control group, LiCl was replaced by (CH_3_)_4_NCl, and the electrolyte composition is 6.82 mol/kg (CH_3_)_4_NCl and 1.64 mol/kg AlCl_3_.

The electrolyte of Cu–Al dual-ion battery is 6.82 mol/kg LiCl, 0.1 mol/kg CuCl and 1.64 mol/kg AlCl_3_, which named as aluminum ion aqueous electrolyte (LiCuAl electrolyte). LiCuAl electrolyte without the addition of LiCl, CuCl or AlCl_3_ are studied as control experiment electrolyte. In addition, CuCl of LiCuAl electrolyte is replaced with CuCl_2_·2H_2_O and named as LiCu^2+^Al electrolyte, which is used in the control group of anode symmetric battery.

### Electrode preparation and Cu–Al dual-ion battery assembly

To prepare a CuS coated titanium foil electrode, 2 mg of CuS sample was mixed with 15 μL of 5 wt.% Nafion solutions and dispersed in 400 uL of ethanol. The mixture was sonicated for over 15 min to form a homogenous dispersion. The dispersion was coated onto a piece of titanium foil (1 × 1 cm^2^) and dried, then a CuS electrode (2 mg/cm^2^ of CuS loading) was obtained. As shown in the SEM image of the electrode cross-section (Fig. S15), the CuS electrode sheet shows a relatively uniform thickness of about 23.4 μm.

For each battery, a piece of copper foil (1 × 1 cm^2^) and a piece of titanium foil (1 × 1 cm^2^) coated with CuS (2 mg/cm^2^ of CuS loading) is used as anode and cathode, respectively. The anode and cathode are separated with GF/D glass fiber separator, then wrapped by aluminum-plastic film. Both the cathode and anode electrodes extend outside of aluminum plastic film through the titanium tab. 400 μL of electrolyte is placed between the electrodes and ensure that the GF/D glass fiber separator is completely wet, then sealed by the aluminum-plastic film.

### Materials characterization

The X-ray diffraction (XRD) patterns of materials were collected by a Shimadzu XRD-6000 diffractometer with Cu Kα radiation (λ = 1.5406 Å) at 40 kV and 40 mA. The scanning range and rate were set to be 3–80° and 10°/min, respectively. The morphology of materials was analyzed by scanning electron microscope (SEM, Zeiss Supra 55). A Renishaw inVia Raman spectrometer was used to measure the Raman spectra of CuS samples and analyze the chemical structure of the samples. The laser source is a He–Ne laser with a wavelength of 633 nm, the laser power was set to 3 mW, and the scanning range was set to 100–1000 cm^−1^. The crystal structure was analyzed by high-resolution transmission electron microscopy (HRTEM, JEM 2100F). The existing forms of constituent elements of samples were analyzed by X-ray photoelectron spectrometer (XPS, Thermo ESCALAB 250XI). ^27^Al nuclear magnetic resonance (NMR) spectra were recorded by a Bruker AV600 NMR spectrometer at 600 MHz with 1.1 M of Al(NO_3_)_3_ in D_2_O as reference.

### Electrochemical measurements

The electrochemical measurements were performed on an electrochemical workstation (CHI660E, Shanghai Chenhua). CV tests of three-electrode system were carried out with CuS electrode as the work electrode, Pt wire as the counter electrode, Ag/AgCl electrode as the reference electrode, and LiAl as the electrolyte (0–1.4 V, 1–100 mV/s). CV tests of CuS cathode electrode symmetrical battery were carried out using a two-electrode system with CuS electrodes and LiAl electrolyte (− 1.5 to 1.5 V, 1 mV/s). GCD tests of copper negative electrode symmetrical battery were carried out using a two-electrode system with copper foil as electrodes, and LiCuAl and LiCu^2+^Al as electrolyte respectively (− 1 to 1 V, 10 mA/cm^2^).

Electrochemical measurements of soft-packed Cu–Al dual-ion battery were carried out using a two-electrode system with CuS electrode as the work electrode, copper foil as the counter electrode and the LiCuAl as the electrolyte (0–1.2 V and 1–100 mV/s for CV tests, 0–1 V for GCD tests). EIS was carried out by applying an open circuit voltage with 1.05 V in the frequency range from 100 kHz to 0.01 Hz. The above of electrochemical measurements were also conducted when LiCuAl without the addition of LiCl, CuCl or AlCl_3_ as electrolyte, respectively.

## Supplementary Information

Supplementary Information.

## Data Availability

The datasets used and/or analysed during the current study available from the corresponding author Y. Wang on reasonable request.
