# Supplementary material for: A high-performance Cu–Al dual-ion battery realized by high-concentration Cl− electrolyte and CuS cathode
Source: Sci Rep. 2022 Nov 4;12:18714. doi: 10.1038/s41598-022-23494-1 (PMC9636194; doi:10.1038/s41598-022-23494-1)
Supplement: Supplementary file 1 — Supplementary Information. [file 41598_2022_23494_MOESM1_ESM.docx]

A High-performance Cu-Al Dual-ion Battery Realized by High-concentration Cl^-^ Electrolyte and CuS Cathode

Meina Tan,^a,1^ Yang Qin,^a,b,1^ Yiping Wang,^a,^* Fazhi Zhang,^a^ Xiaodong Lei^a,^*

^a^State Key Laboratory of Chemical Resource Engineering, Beijing University of Chemical Technology, Beijing 100029, China

^b^Advanced Technology Department, RiseSun MGL, Inc. Beijing 102299, China

Correspondence and requests for materials should be addressed to Y. Wang (email: [wangyiping@buct.edu.cn](mailto:wangyiping@buct.edu.cn)) or X. Lei (email: [leixd@mail.buct.edu.cn](mailto:leixd@mail.buct.edu.cn))

^1^ These authors contributed equally to this work

**Supplementary Information**


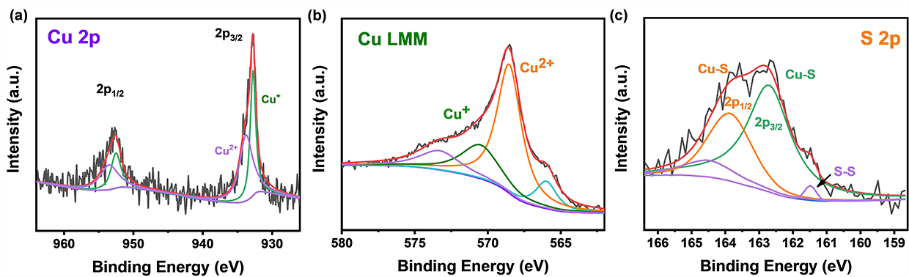


**Figure S1.** High-resolution XPS spectra of (a) Cu 2p, (b) Cu LMM and (c) S 2p in CuS sample.


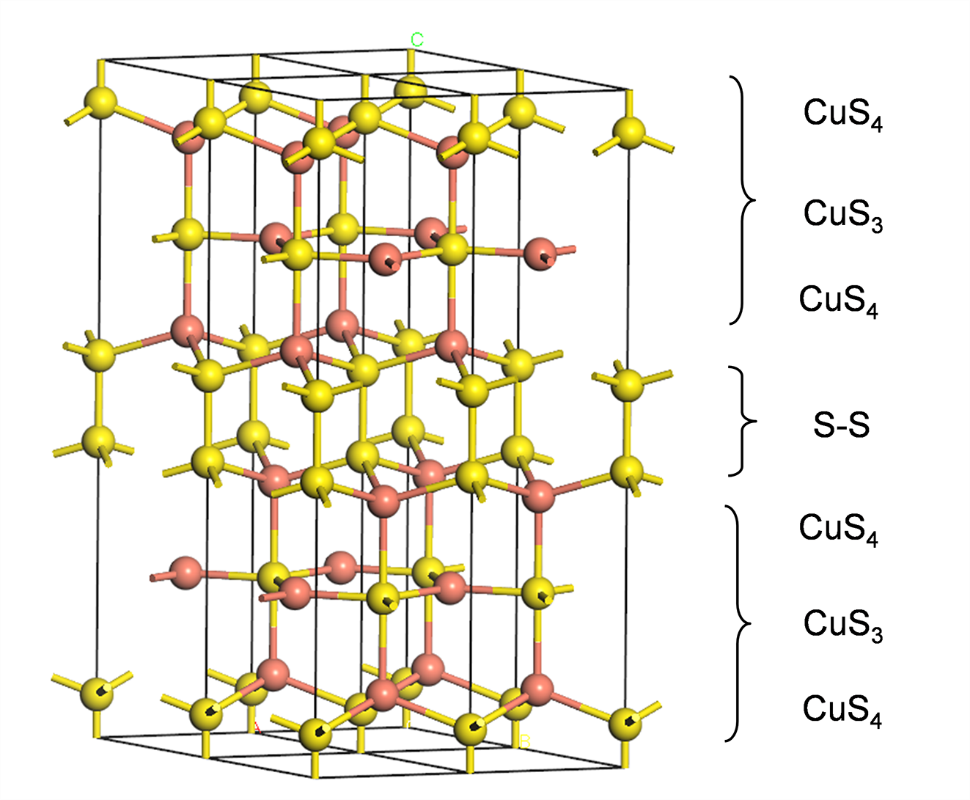


**Figure S2.** Crystal structure of covellite CuS.


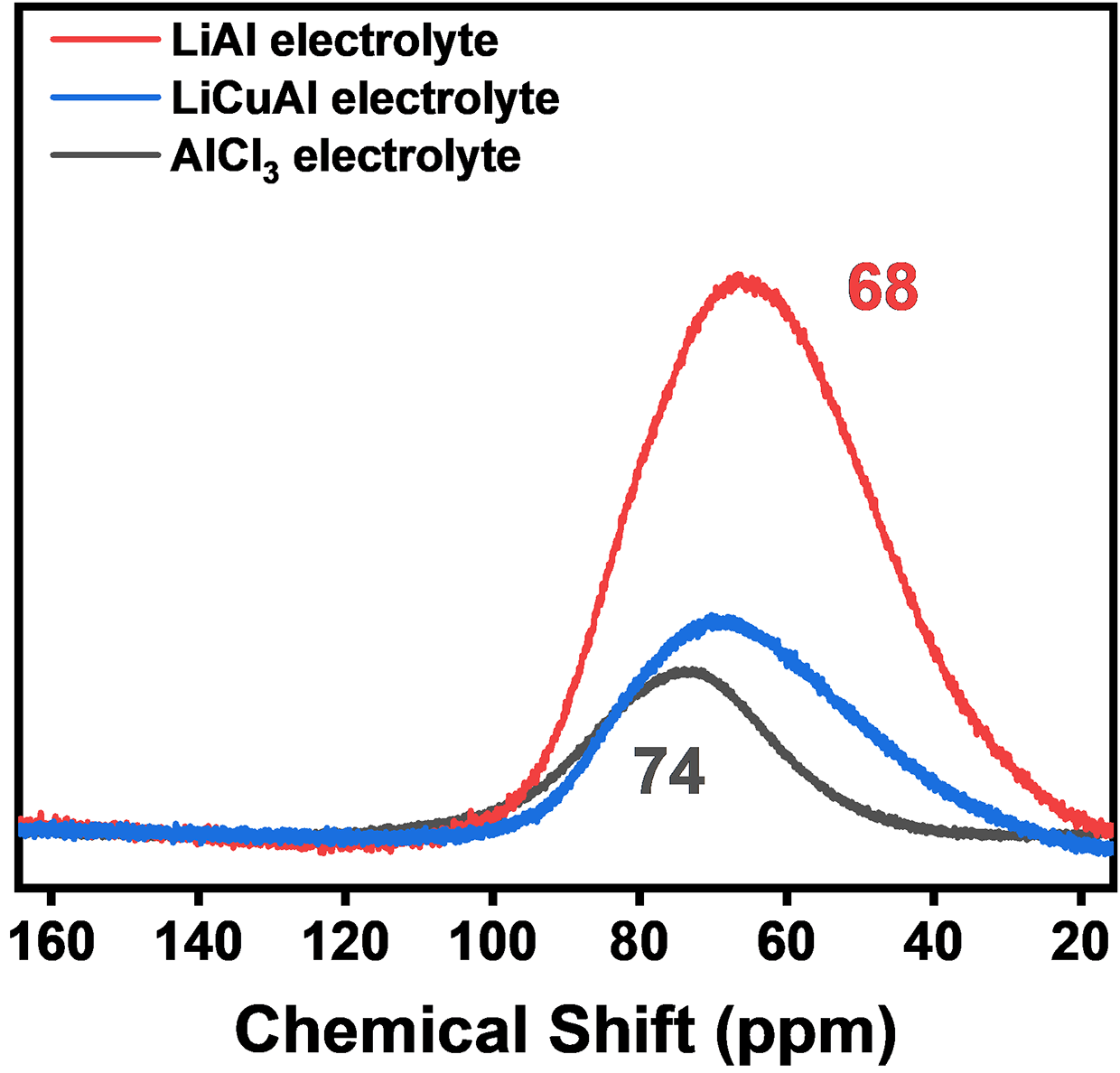


**Figure S3.** ^27^Al NMR spectra of electrolytes containing Al species.


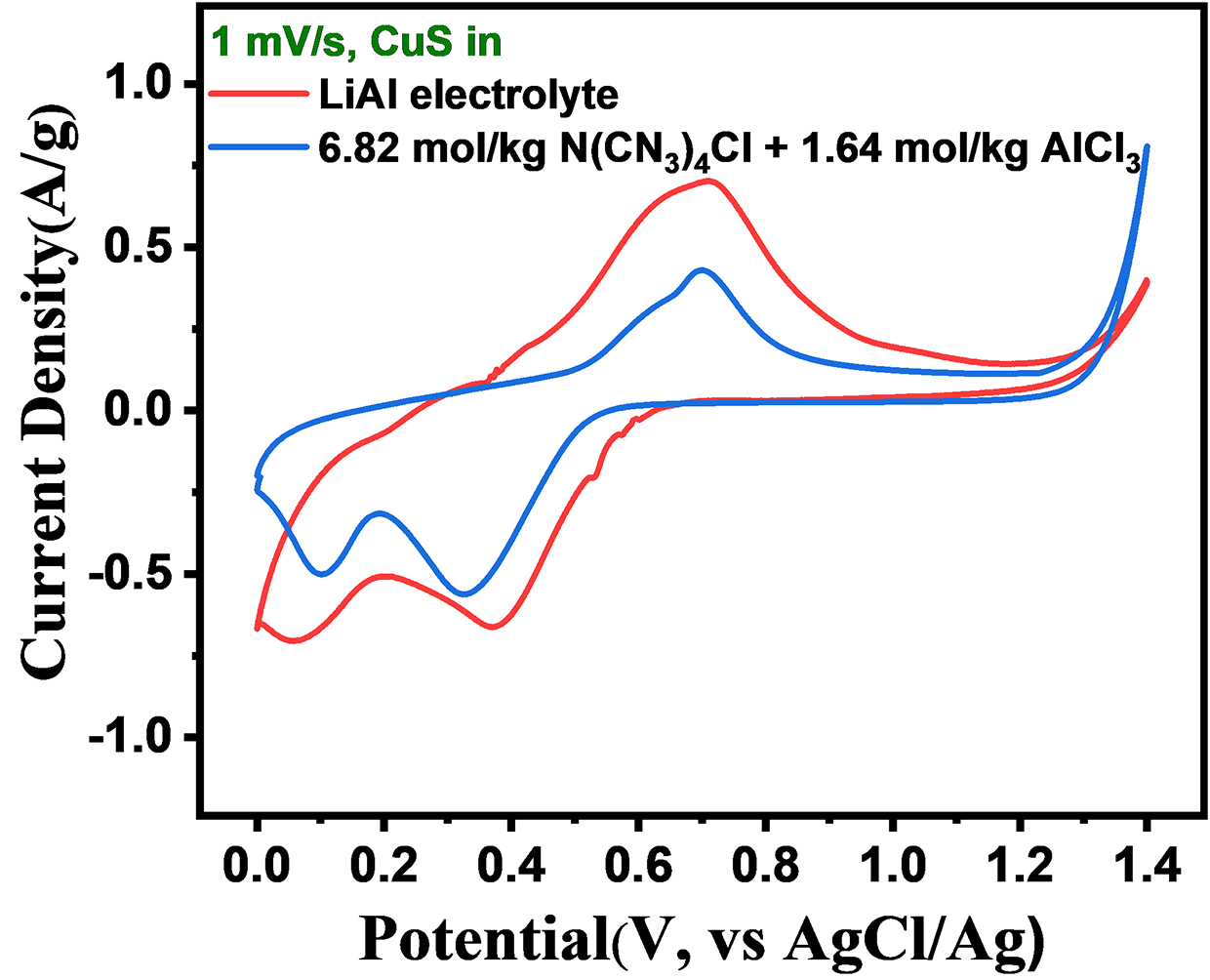


**Figure S4.** CV curves of 3-electrode system measured in LiAl and (CH_3_)_4_NCl-based electrolytes.

**
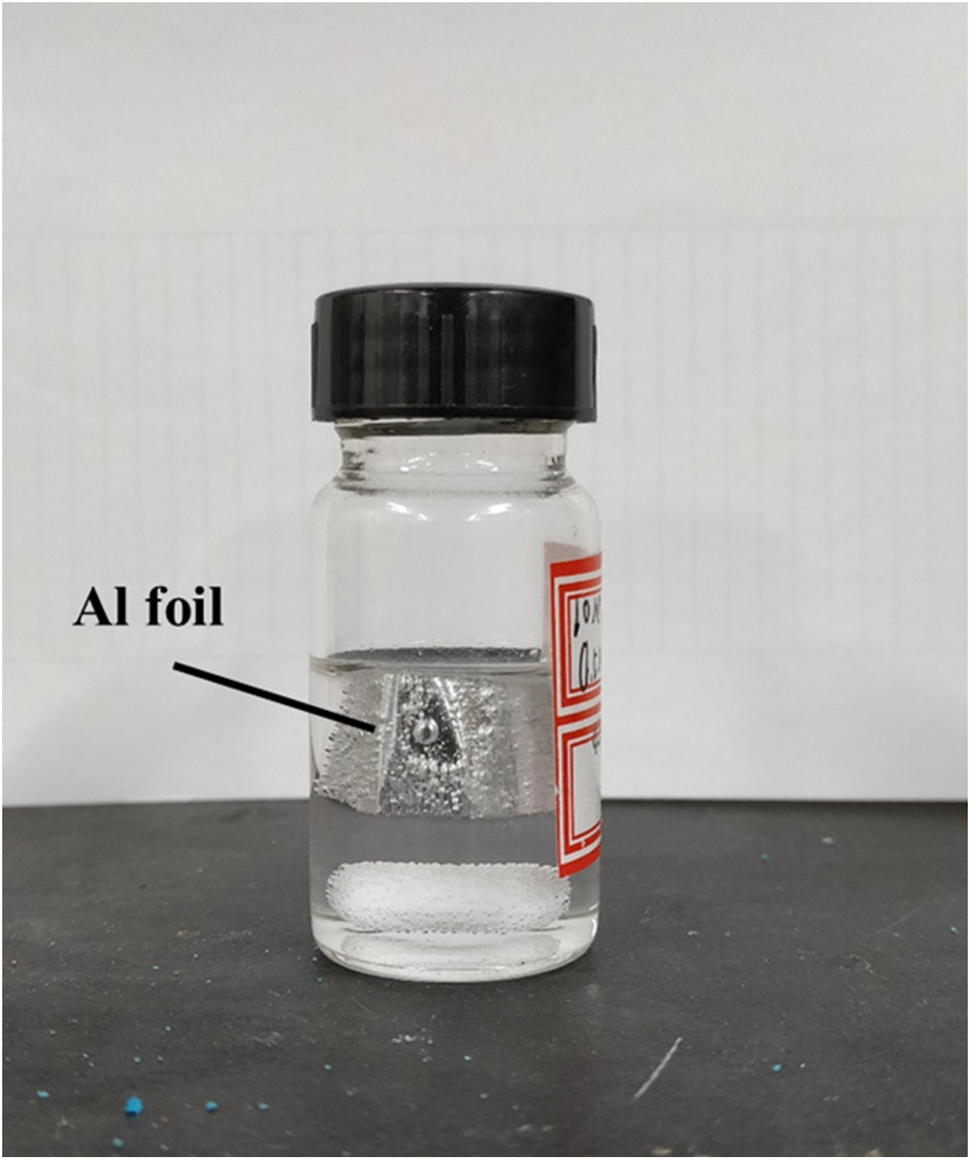
**

**Figure S5.** Self-corrosion of Al foil in LiAl electrolyte.


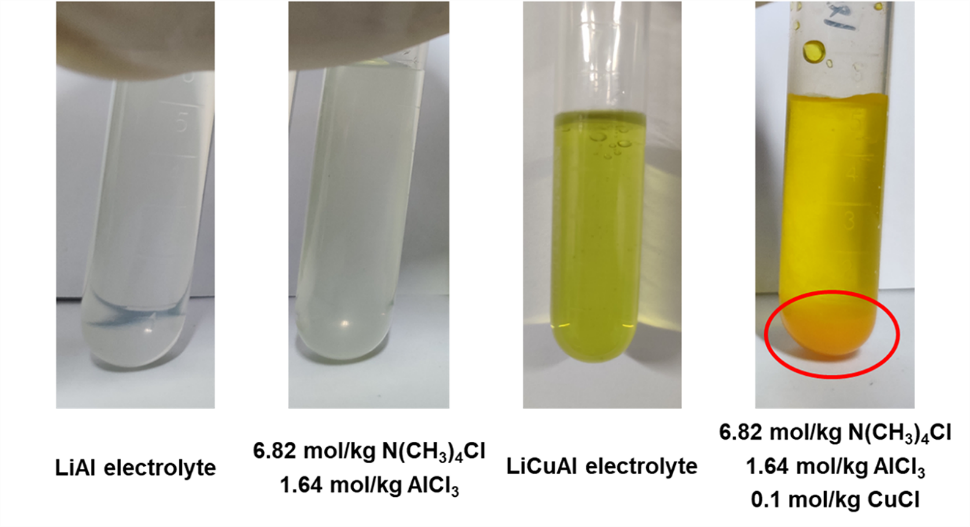


**Figure S6.** Photos of different electrolytes.


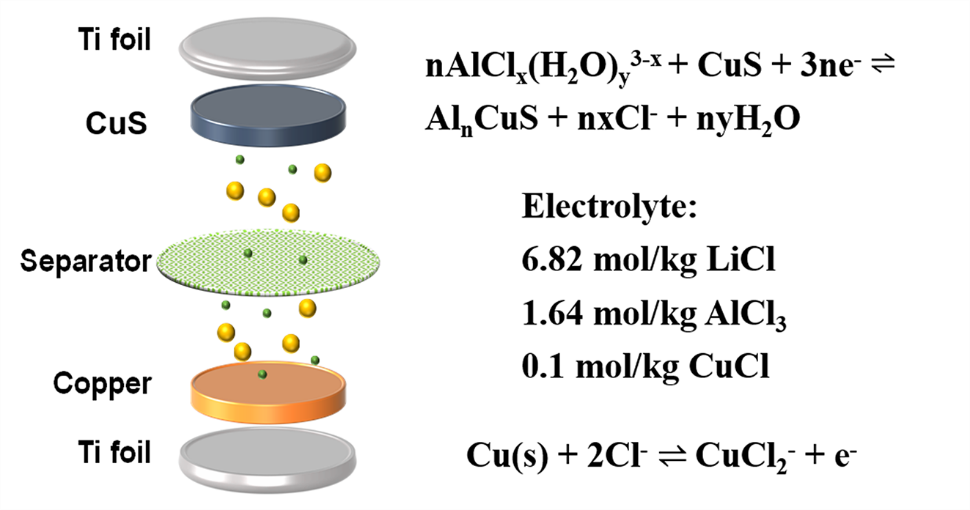


**Figure S7.** Scheme of soft-pack Cu-Al dual-ion battery.


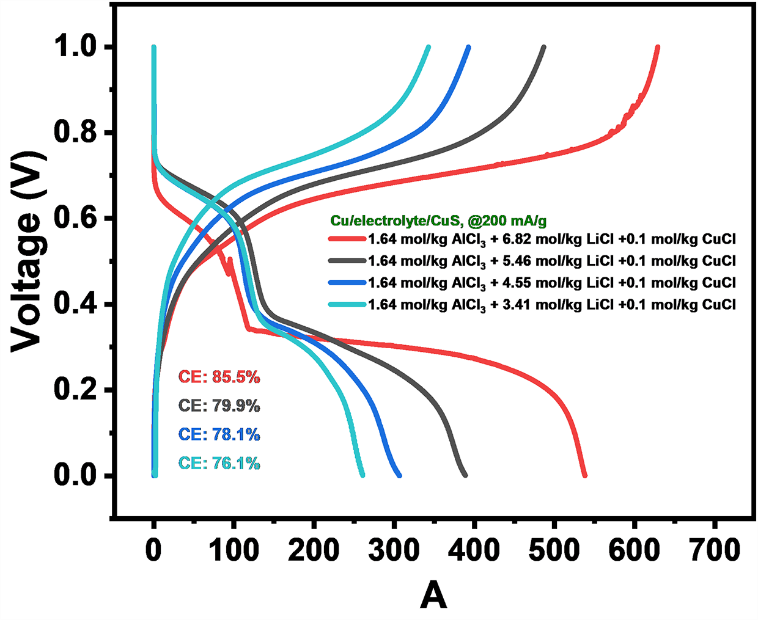


**Figure S8.** GCD curves of Cu-Al dual-ion batteries in electrolytes with different chloride ion concentrations.

**
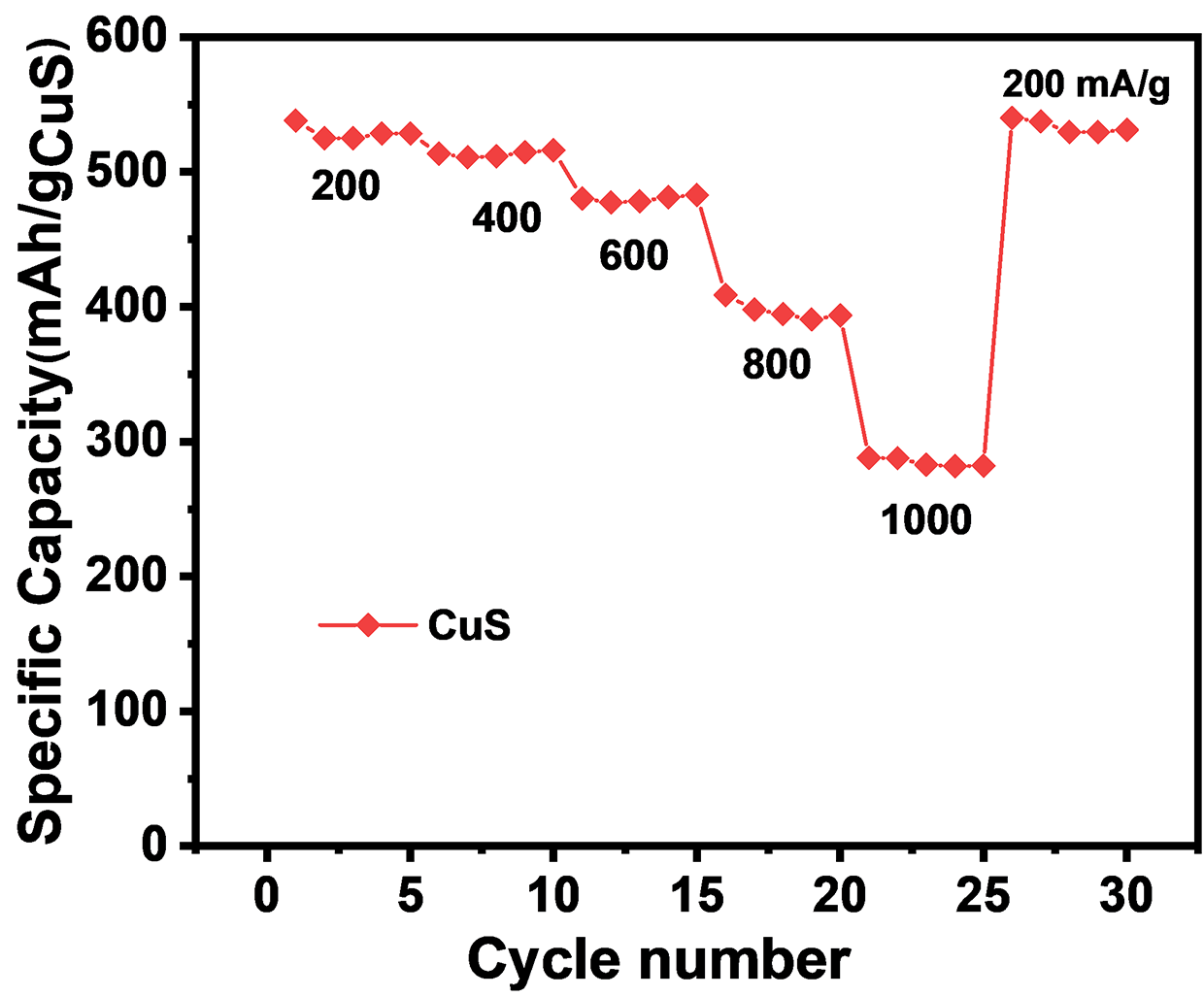
**

**Figure S9.** The charge/discharge performances of Cu-Al dual-ion battery at various current rates.

**
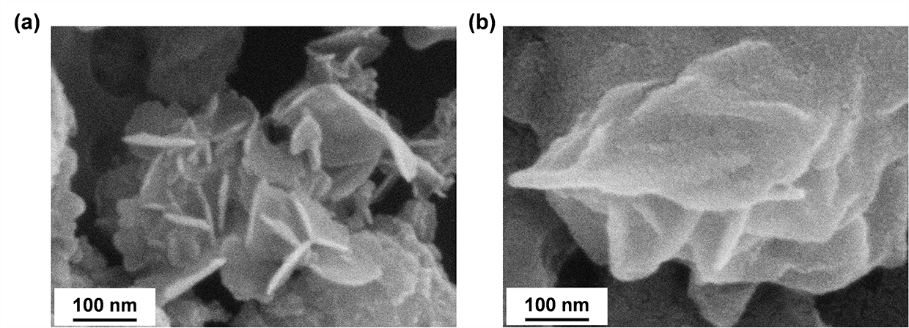
**

**Figure S10.** SEM images of CuS before (a) and after (b) cycling test.


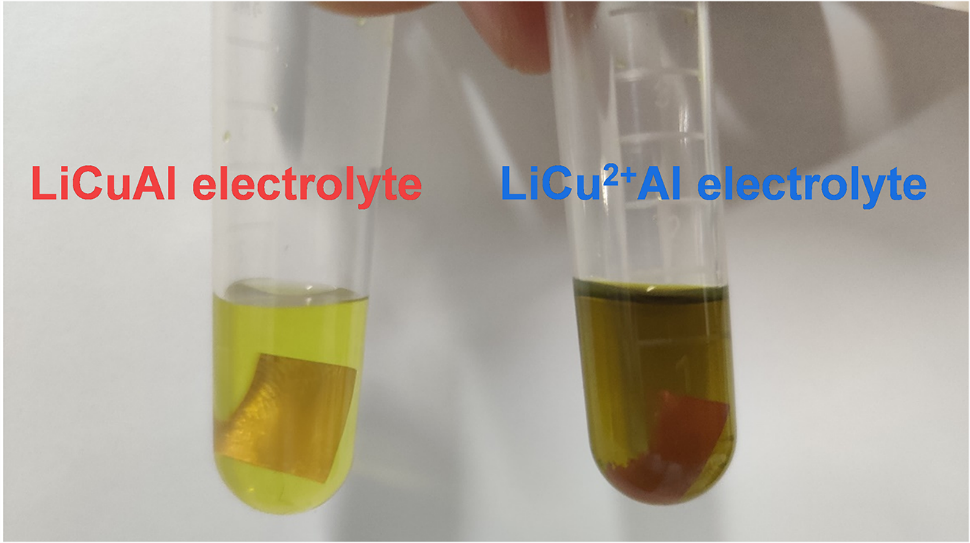


**Figure S11.** Overnight state of copper foil in different electrolytes.

**
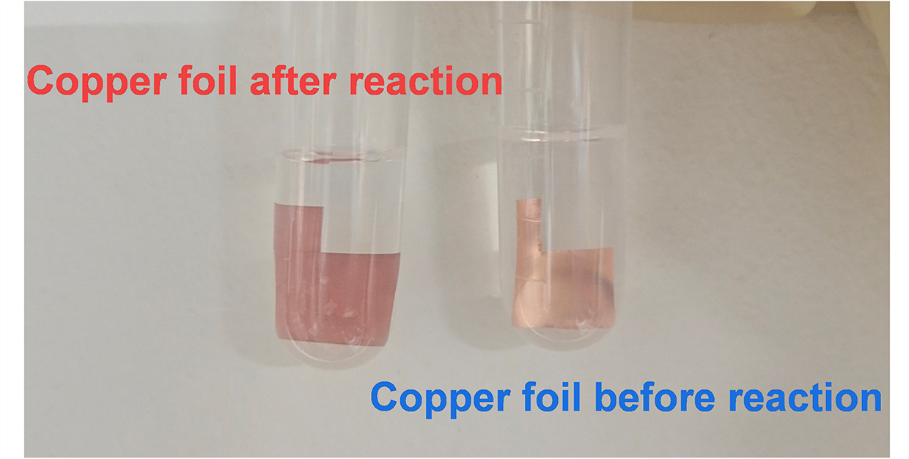
**

**Figure S12.** Changes of copper foil before and after reaction in LiCuAl electrolyte.

**
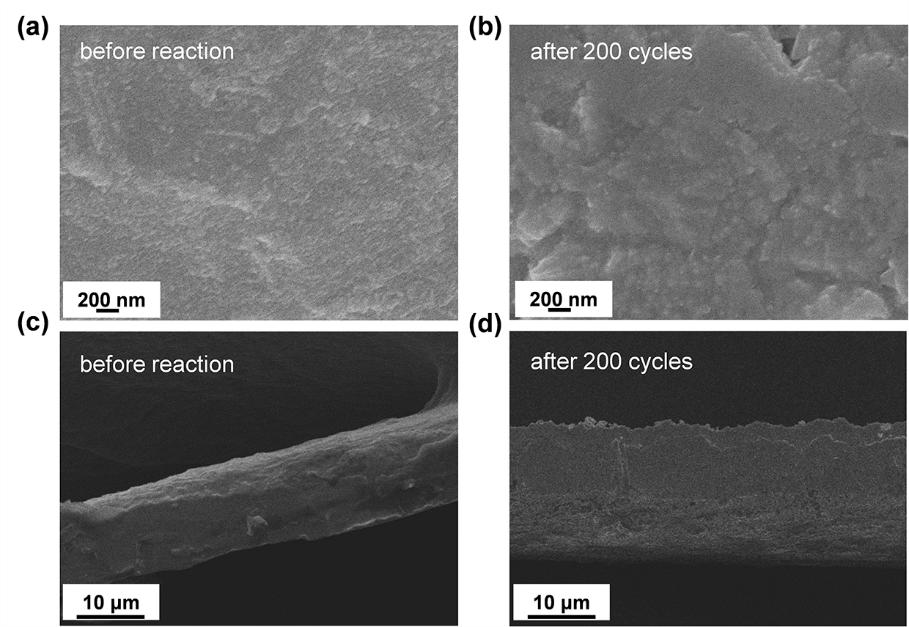
**

**Figure S13.** SEM images of (a and b) upper surface and (c and d) cross-section of copper before and after the cycling reaction.

**
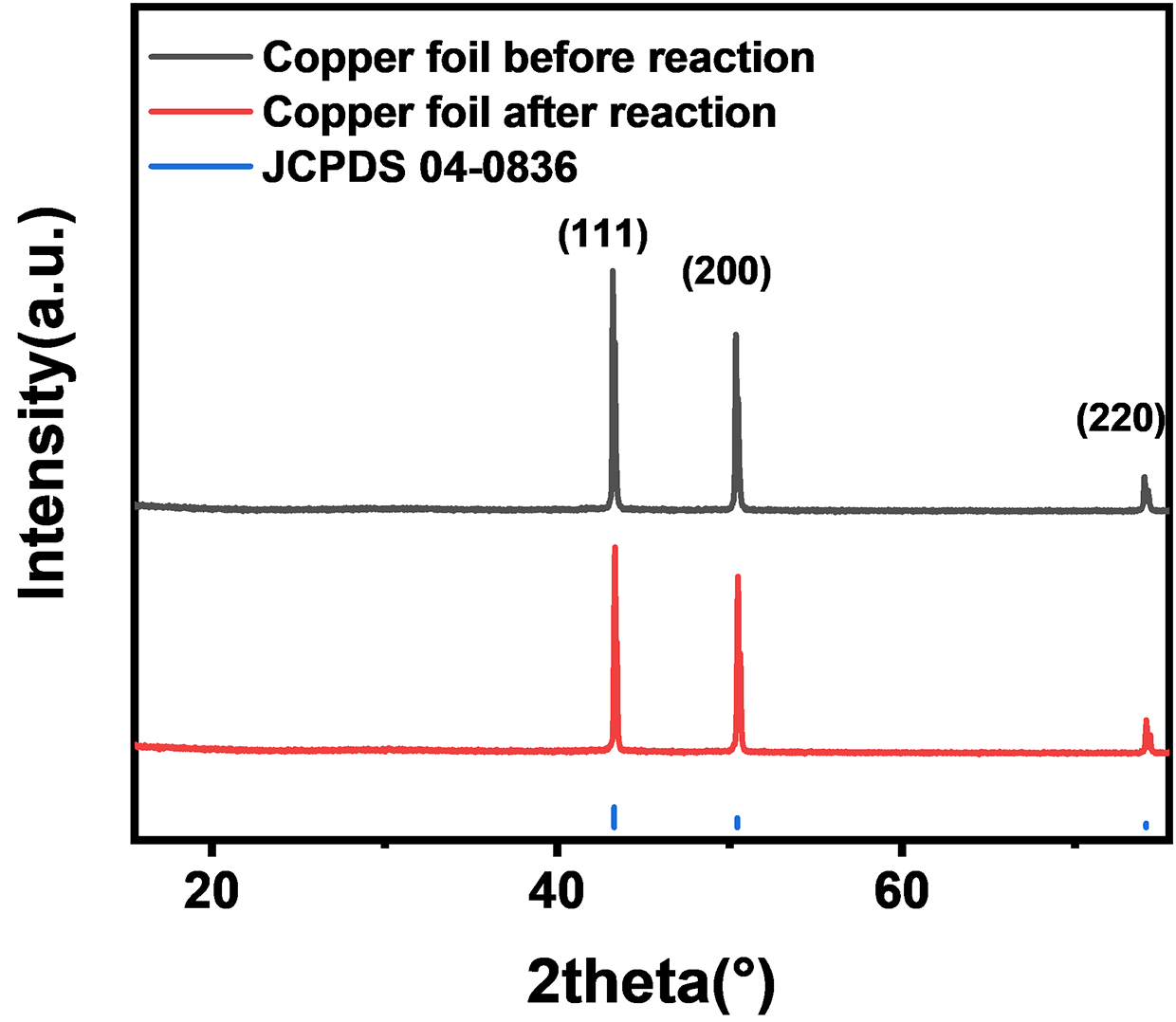
**

**Figure S14.** XRD of Cu foil before and after cycling test.

**
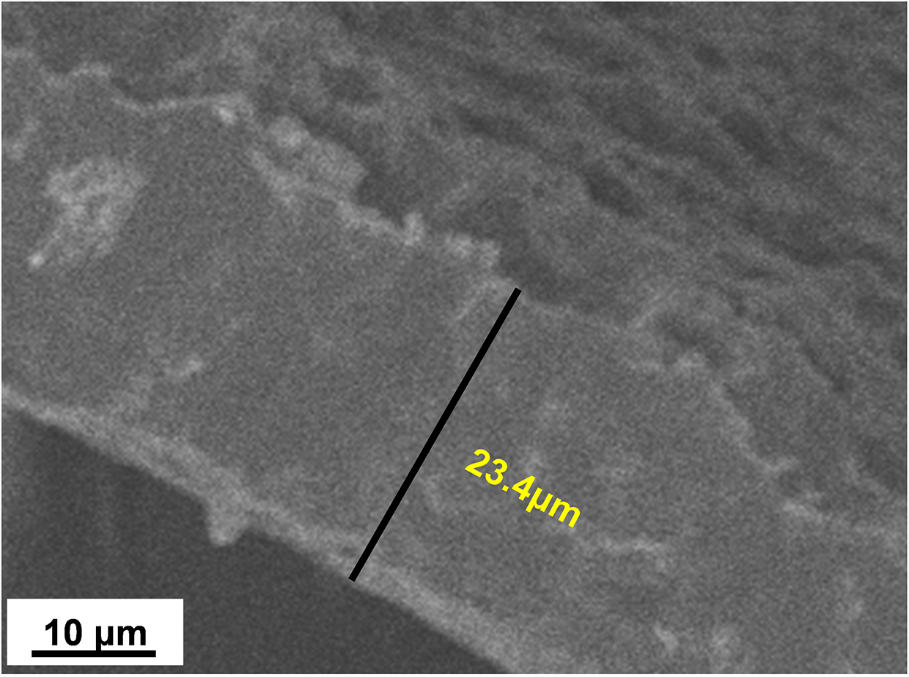
**

**Figure S15.** SEM image of cross-section view of CuS electrode.

**Table S1. Positive electrode materials in aqueous and other electrolytes for AIBs.**

Positive electrode material, electrolyte, specific capacity, initial capacity and capacity retention.

| Positive electrode material | Electrolyte | Specific capacity  (mAh g^−1^ ) | Initial Capacity  (mAh g^−1^ ) | Capacity retention  (%) | Ref. |
| --- | --- | --- | --- | --- | --- |
| CuS | AlCl_3_: LiCl: CuCl | 538 at 200 mA g^−1^ | 259 at 1000 mA g^−1^ | 88.6 after 200 cycles at 1000 mA g^−1^ | This work |
| SWCNT | WIS-AlCl_3_ | 790 at 5000 mA g^−1^ | 600 at 1000 mA g^−1^ | ~100 after 200 cycles at 1000 mA g^−1^ | ^1^ |
| Al_x_MnO_2_ | 2 M Al(OTF)_3_ | 100 at 3000 mA g^−1^ | ~680 at 100 mA g^−1^  ~350 at 1000 mA g^−1^ | 67.6 after 80 cycles at 100 mA g^−1^;  84 after 20 cycles at 1000 mA g^−1^ | ^2^ |
| Carbon paper (Liquid Ga Negative Electrode ) | NaCl-AlCl_3_ | 139.8 at 1000 mA g^−1^ | 139.8 at 1000 mA g^−1^ | 90.8 after 1200 cycles at 1000 mA g^−1^ | ^3^ |
| K_2_CuFe(CN)_6_ | 1 M Al_2_(SO_4_)_3_ | 167.7 at 1000 mA g^−1^ | 53.2 at 500 mA g^−1^ | 89.1 after 100 cycles at 500 mA g^−1^ | ^4^ |
| CuHCF | 0.5 M Al_2_(SO_4_)_3_ | 62.9 at 50 mA g^−1^ | 46.9 at 400 mA g^−1^ | 54.9 after 1000 cycles at 400 mA g^−1^ | ^5^ |
| PBA FeFe(CN)_6_ | 5 M Al(OTF)_3_ | 116 at 150 mA g^−1^ | 116 at 150 mA g^−1^ | 60.8 after 100cycles at 150 mA g^−1^ | ^6^ |
| PZ | 5 M Al(OTF)_3_ | 41 at 200 mA g^−1^ | 132 at 50 mA g^−1^ | 76.5 after 300 cycles at 50 mA g^−1^ | ^7^ |

**Table S2. Positive electrode materials in the organic system for AIBs.**

Positive electrode material, electrolyte, specific capacity, initial capacity and capacity retention.

| Positive electrode material | Electrolyte | Specific capacity  (mAh g^−1^ ) | Initial Capacity  (mAh g^−1^ ) | Capacity retention  (%) | Ref. |
| --- | --- | --- | --- | --- | --- |
| CuS | AlCl_3_: LiCl: CuCl | 538 at 200 mA g^−1^ | 259 at 1000 mA g^−1^ | 88.6 after 200 cycles at 1000 mA g^−1^ | This work |
| G-VS_2_ | 1.3 : 1 AlCl_3_: [EMIm]Cl | 493 at 20 mA g^−1^ | 186 at 100 mA g^−1^ | 47.5 after 50 cycles at 100 mA g^−1^ | ^8^ |
| NiS nanobelts | 1.3 : 1 AlCl_3_: [EMIm]Cl | 104.7 at 200 mA g^−1^ | 104.7 at 200 mA g^−1^ | 90 after 100 cycles at 200 mA g^−1^ | ^9^ |
| CuS@C | 1.3 : 1 AlCl_3_: [EMIm]Cl | 240 at 20 mA g^−1^ | 240 at 20 mA g^−1^ | 37.5 after 100 cycles at 20 mA g^−1^ | ^10^ |
| G-SnS_2_ | 1.3 : 1 AlCl_3_: [EMIm]Cl | 112 at 1000 mA g^−1^ | 392 at 100 mA g^−1^ | 79.1 after 5 cycles at 100 mA g^−1^ | ^11^ |
| Co_9_S_8_@CNT–CNF | 1.3 : 1 AlCl_3_: [EMIm]Cl | 315 at 100 mA g^−1^ | 154 at 1000 mA g^−1^ | 56.5 after 6000 cycles at 1000 mA g^−1^ | ^12^ |
| V_2_O_5_ nano-wires | 1.1 : 1 AlCl_3_: [EMIm]Cl | 305 at 125 mA g^−1^ | 305 at 125 mA g^−1^ | 89.5 after 20 cycles at 125  mA g^−1^ | ^13^ |
| Ni_2_P/rGO | 1.1 : 1 AlCl_3_: [EMIm]Cl | 274.5 at 100 mA g^−1^ | 274.5 at 100mA g^−1^;  187.4 at 200 mA g^−1^ | 26.6 after 500 cycles at 100  mA g^−1^  ;32.5 after 3000 cycles at 200  mA g^−1^ | ^14^ |
| EG | 1.1 : 1 AlCl_3_: [EMIm]Cl | 70 at 100 mA g^−1^ | 88 at 20 mA g^−1^ | 79.5 after 600 cycles at 20  mA g^−1^ | ^15^ |
| MoS_2_/CNFs | 1.1 : 1 AlCl_3_: [EMIm]Cl | 293.2 at 100 mA g^−1^ | 293.2 at 100 mA g^−1^ | 43.2 after 200 cycles at 100  mA g^−1^ | ^16^ |
| GF | 1.1 : 1 AlCl_3_: [EMIm]Cl | 120 at 100 mA g^−1^ | 115 at 50 mA g^−1^ | ~100 after 1000 cycles at 50  mA g^−1^ | ^17^ |
| Sulfur-carbon composite (S–C) | 1.3 : 1 AlCl_3_: [EMIm]Cl | 1320 at 50 mA g^−1^ | 1300 at 50 mA g^−1^ | 76 after 22 cycles at 50 mA g^−1^ | ^18^ |
| S@CNF paper | 1.3 : 1 AlCl_3_: [EMIm]Cl | 1250 at 83.75 mA g^−1^ | 1250 at 83.75 mA g^−1^ | 50 after 10 cycles at 83.75 mA g^−1^ | ^19^ |

**Supplementary Note 1**

**Detailed analysis of the Eq. (1)-(3)**

Eq. (1) shows the hydrolysis reaction of aluminum ions in the aqueous solution. Eq. (2) shows the chemical equilibrium of the aluminum foil in the aqueous solution containing aluminum ions. When the aqueous solution contains a high concentration of Cl^-^, the reaction involved in Eq. (3) proceeds in the direction of AlCl_x_(H_2_O)_y_^3-x^ formation, resulting in the decrease of Al^3+^ concentration in the solution. According to Le Chatelier’s principle, the equilibrium of reaction involved in Eq. (2) shifts to the right, resulting in spontaneous corrosion of the Al foil in the LiAl electrolyte.

**Supplementary References**

1. Pan, W. et al. High‐energy swcnt cathode for aqueous Al‐ion battery boosted by multi‐ion intercalation chemistry. Adv. Energy Mater. 11, 2101514 (2021).

2. Yan, C. et al. Architecting a stable high-energy aqueous Al-ion battery. J. Am. Chem. Soc. 142, 15295-15304 (2020).

3. Wang, J. et al. Stable interface between a NaCl-AlCl_3_ melt and a liquid Ga negative electrode for a long-life stationary Al-ion energy storage battery. ACS Appl. Mater. Interfaces. 12, 15063-15070 (2020).

4. Yan, L. et al. 9,10-anthraquinone/K_2_CuFe(CN)_6_: A highly compatible aqueous aluminum-ion full-battery configuration. ACS Appl. Mater. Interfaces. 13, 8353-8360 (2021).

5. Liu, S. et al. Copper hexacyanoferrate nanoparticles as cathode material for aqueous Al-ion batteries. J. Mater. Chem. A. 3, 959-962 (2015).

6. Zhou, A. et al. Water-in-salt electrolyte promotes high-capacity FeFe(CN)_6_ cathode for aqueous Al-ion battery. ACS Appl. Mater. Interfaces. 11, 41356-41362 (2019).

7. Chen, J. et al. Rechargeable aqueous aluminum organic batteries. Angew. Chem. Int. Ed. Engl. 60, 5794-5799 (2021).

8. Wu, L. et al. A rechargeable aluminum-ion battery based on a VS_2_ nanosheet cathode. Phys. Chem. Chem. Phys. 20, 22563-22568 (2018).

9. Yu, Z. et al. Hexagonal NiS nanobelts as advanced cathode materials for rechargeable Al-ion batteries. Chem Commun (Camb). 52, 10427-10430 (2016).

10. Wang, S. et al. High-performance aluminum-ion battery with CuS@C microsphere composite cathode. ACS Nano. 11, 469-477 (2017).

11. Hu, Y. et al. An innovative freeze-dried reduced graphene oxide supported SnS_2_ cathode active material for aluminum-ion batteries. Adv. Mater. 29, 1606132 (2017).

12. Hu, Y. et al. A binder-free and free-standing cobalt sulfide@carbon nanotube cathode material for aluminum-ion batteries. Adv. Mater. 30, 1703824 (2018).

13. Jayaprakash, N. et al. The rechargeable aluminum-ion battery. Chem Commun (Camb). 47, 12610-12612 (2011).

14. Tu, J. et al. Nickel phosphide nanosheets supported on reduced graphene oxide for enhanced aluminum-ion batteries. ACS Sustainable Chem. Eng. 7, 6004-6012 (2019).

15. Wang, S. et al. A novel dual-graphite aluminum-ion battery. Energy Storage Mater. 12, 119-127 (2018).

16. Yang, W. et al. Flexible free-standing MoS_2_/carbon nanofibers composite cathode for rechargeable aluminum-ion batteries. ACS Sustainable Chem. Eng. 7, 4861-4867 (2019).

17. Li, N. et al. High-performance wire-shaped aluminum ion batteries based on continuous graphene fiber cathodes. J. Power Sources. 488, 229460 (2021).

18. Gao, T. et al. A rechargeable Al/S battery with an ionic-liquid electrolyte. Angew. Chem. Int. Ed. Engl. 55, 9898-9901 (2016).

19. Yu, X. et al. Electrochemical energy storage with a reversible nonaqueous room‐temperature aluminum–sulfur chemistry. Adv. Energy Mater. 7, 1700561 (2017).
